# Trabecular bone deficits predominate in the appendicular skeleton of midlife women living with HIV: findings from a cross-sectional study in Zimbabwe

**DOI:** 10.1093/jbmr/zjaf021

**Published:** 2025-01-25

**Authors:** Mícheál Ó Breasail, Tafadzwa Madanhire, Cynthia Kahari, Peter R Ebeling, Victoria Simms, Lisa K Micklesfield, Rashida A Ferrand, Celia L Gregson, Kate A Ward

**Affiliations:** Department of Medicine, Faculty of Medicine, Nursing and Health Sciences, School of Clinical Sciences, Monash Medical Centre, Monash University, Clayton, Victoria, 3168, Australia; Population Health Sciences, Bristol Medical School, Bristol BS8 1NU, United Kingdom; The Health Research Unit Zimbabwe, Biomedical Research and Training Institute, Harare, Zimbabwe; Department of Infectious Disease Epidemiology, Faculty of Epidemiology and Population Health, London School of Hygiene and Tropical Medicine, London WC1E 7HT, United Kingdom; The Health Research Unit Zimbabwe, Biomedical Research and Training Institute, Harare, Zimbabwe; Department of Infectious Disease Epidemiology, Faculty of Epidemiology and Population Health, London School of Hygiene and Tropical Medicine, London WC1E 7HT, United Kingdom; Department of Medicine, Faculty of Medicine, Nursing and Health Sciences, School of Clinical Sciences, Monash Medical Centre, Monash University, Clayton, Victoria, 3168, Australia; The Health Research Unit Zimbabwe, Biomedical Research and Training Institute, Harare, Zimbabwe; Department of Infectious Disease Epidemiology, Faculty of Epidemiology and Population Health, London School of Hygiene and Tropical Medicine, London WC1E 7HT, United Kingdom; SAMRC/Wits Developmental Pathways for Health Research Unit, Department of Paediatrics, Faculty of Health Sciences, School of Clinical Medicine, University of the Witwatersrand, Johannesburg, Johannesburg, South Africa; The Health Research Unit Zimbabwe, Biomedical Research and Training Institute, Harare, Zimbabwe; Clinical Research Department, Faculty of Infectious and Tropical Diseases, London School of Hygiene and Tropical Medicine, London WC1E 7HT, United Kingdom; The Health Research Unit Zimbabwe, Biomedical Research and Training Institute, Harare, Zimbabwe; Global Musculoskeletal Research Group, Musculoskeletal Research Unit, Bristol Medical School, University of Bristol, Bristol BS10 5NB, United Kingdom; MRC Lifecourse Epidemiology Centre, Human Development and Health, University of Southampton, Southampton SO16 6YD, United Kingdom; MRC Unit, The Gambia @ London School of Hygiene and Tropical Medicine, Banjul, The Gambia

**Keywords:** analysis/quantitation of bone, analysis/quantitation of bone, aging, epidemiology, menopause

## Abstract

HIV-related mortality has fallen due to the scale-up of antiretroviral therapy (ART), so more women living with HIV (WLH) now live to reach menopause. Menopausal estrogen loss causes bone loss, as do HIV and certain ART regimens. However, quantitative bone data from WLH are few in Africa. A cross-sectional study of women aged 40-60 yr (49% WLH) was conducted in Harare, Zimbabwe. Menopause status, fracture history, HIV status and treatment, and anthropometry were collected, and radial/tibial peripheral QCT (pQCT) scans were performed. pQCT outcomes were distal radius and tibia trabecular volumetric BMD (vBMD), total area, and compressive bone strength (BSIc); proximal radius and tibia cortical vBMD, BMC, cortical thickness, bone area, and stress–strain index (SSI). Linear regression determined differences by HIV status, minimally adjusted for age and menopause status, and further adjusted for height and fat mass. Relationships between pQCT parameters and major osteoporotic fracture history were explored using univariate logistic regression. In WLH, linear regression assessed associations between HIV and ART durations on pQCT measures. 384 women mean (SD) age 49.7 (5.8) yr had pQCT data. WLH had lower absolute pQCT measures at all sites. Overall, HIV-related deficits were robust to adjustment for age, menopause status, height, and fat mass: WLH had lower trabecular vBMD (radius −7.3 [−12.5; −2.0]%, tibia −5.4 [−9.1; −1.7]%), and cortical vBMD (radius −3.5 [−5.9; −1.1]%, tibia −1.1 [−1.6; −0.5]%). Strength estimates were lower in WLH and of similar magnitude at the radius and tibia. Longer HIV duration was associated with lower radius bone area, BMC, and estimates of bone strength, independent of ART duration. Trabecular deficits predominate in WLH, though with age cortical compartment bone loss may increase in importance. This is particularly concerning as these differences were observed at the radius, a common site of postmenopausal osteoporotic fracture.

## Introduction

The global burden of HIV disproportionally impacts Africa, with the greatest number of people with HIV living in East and Southern Africa.^1^ In recent decades the widespread availability of antiretroviral therapy (ART) has dramatically reduced HIV-related mortality,^2^ improving life expectancy across the region. Hence, HIV is now a chronic disease of aging, characterized by age-associated non-infectious complications, such as osteoporosis and other non-communicable diseases (NCDs).^3–5^ While data are limited, there is growing evidence to suggest that HIV infection, its treatment, and underlying associated sociodemographic risk factors all negatively influence bone health. However, much of this literature is drawn from men in high-income countries.^6–8^ In contrast, relatively few data are available for African women at midlife, typically defined as ages 40-60,^9^ a critical period for future bone health.^10–12^

Data from urban South Africa have suggested that women living with HIV (WLH) have deficits in bone even prior to entering the menopause transition,^13^ and more recent South African and Zimbabwean data have highlighted that independent of age, WLH have lower DXA measured areal BMD,^14^^,^^15^ lose more bone mineral during menopause,^14^ and have both a higher prevalence of *T*-score defined osteoporosis and previous major osteoporotic fractures compared to HIV negative women.^15^ Taken together, African WLH may be at higher risk of osteoporosis and fracture at midlife, earlier in the lifecourse than WLH in high-income settings,^12^ potentially exacerbated by subsequent menopausal-related bone loss.^16^

While DXA remains the clinical gold standard for defining osteoporosis, it is a 2D modality measuring areal BMD and is unable to provide compartment-specific estimates of volumetric BMD (vBMD), distribution, or strength. In contrast, peripheral QCT (pQCT) measures vBMD, mineral content, geometry, and strength estimates in the appendicular skeleton. We have previously published pQCT data from an urban South African cohort of midlife Black women (*n* = 430, 18% WLH) and observed that absolute BMD and strength were progressively lower by menopause stage in both WLH and HIV-negative women, but WLH had lower absolute pQCT values at each menopause stage.^17^ The current cross-sectional study took place in Harare, Zimbabwe, where the prevalence of HIV in women aged 15-64 yr is 16%,^18^ and pQCT was used to obtain measures of volumetric bone density, mass, geometry, and strength in Zimbabwean women between 40 and 60 yr. The study aimed to quantify differences in pQCT parameters between women living with and without HIV, determine which pQCT parameters were associated with a history of prior osteoporotic fracture, and lastly identify HIV-specific factors associated with deficits in bone density, geometry, and strength.

## Material and methods

### Recruitment

Study design and recruitment have been described previously^19^ but briefly, between April and December 2020, women aged 40-60 yr resident in Harare were enrolled in a cross-sectional study, sampled by 4 age strata (40-44, 45-49, 50-54, and 55-60 yr, to span the period of menopausal transition) and HIV status. The aim was to recruit 400 women, equally split by HIV status. WLH were recruited from Sally Mugabe (formerly known as Harare Central) and Parirenyatwa Hospital HIV clinics, the two main public healthcare hospitals in Harare. Women recruited with HIV were asked to identify two female friends of a similar age, with phone access, potentially interested in participating (this approach was necessary to comply with COVID-19 guidelines in place at the time). Those aged between 40 and 60 yr, residents of Harare, not acutely unwell, who were willing to have an HIV test, were recruited.

### Data collection

Socio-demographic data, including highest educational attainment, employment status, lifestyle information (tobacco smoking/use and alcohol intake), and medical and prior fracture history (classed as any fracture or major osteoporotic fracture [MOF], ie, hip, spine, wrist, or humerus), were captured by questionnaire administered by a female nurse. Women currently having regular periods were classified as premenopausal, women having irregular periods and/or a period within the previous 12 mo were classified as perimenopausal, and women who had had no bleeding for 12 mo or more were classified as postmenopausal. Medications were recorded, including ART regimen, menopausal hormone therapy, and any medicines affecting bone health.

### HIV testing

All women recruited without an established HIV diagnosis had a point-of-care HIV antibody test performed using Alere Determine HIV-1/2 (Alere San Diego, Inc.). If negative, they were enrolled in the HIV-negative group. If positive, after a confirmatory test (Chembio SURE CHECK HIV ½ Assay), they were offered enrollment into the WLH group, and all were referred to local HIV services.^20^ GeneXpertTM HIV-1 Viral Load (Cepheid) was used to determine HIV viral load, with a lower limit of detection of 40 copies/mL.

### Anthropometry

Two nurses measured height (cm) and weight (kg) on each participant using a Seca 213 stadiometer and Seca 875 digital scales (Seca Precision for health, Seca Mechanical Floor Scales Class III), respectively, with the mean of both measurements calculated. BMI was calculated (kg/m^2^).

### Peripheral QCT (pQCT) scanning

Scans, at the radius (at 4% and 33% of the limb length proximal to the distal endplate) and tibia (at 4%, and 38% of the limb length proximal to the distal endplate), were acquired on a XCT 2000L (Stratec Medizintechnik) using a voxel size of 0.5 × 0.5 mm and slice thickness of 2 mm. CT scan speed was 30 mm/s and scout view scan speed was 40 mm/s. All pQCT images were processed using the manufacturer’s software (Stratec XCT version 6.2). At distal 4% sites, CALCBD analysis was used to calculate total cross-sectional area (CSA) and total and trabecular vBMD. CALCBD contour mode 1 (ie, the threshold algorithm) was used to exclude pixels in the defined ROI that fell below the default threshold of 180 mg/cm^3^; peel mode 1 (ie, concentric peel) peeled away the outer 55% of the total CSA of the bone with the remaining inner 45% CSA classed as trabecular bone. At proximal cortical-rich sites (33%/38%), CORTBD was used to define cortical vBMD and cortical CSA. The algorithm removes all voxels within the ROI that have an attenuation coefficient below the threshold. The default threshold of 710 mg/cm^3^ was used with separation mode 1. Total CSA was defined at proximal sites using an edge detection threshold of 280 mg/cm^3^. Cortical thickness was calculated using a circular ring model. Stress–strain Index (SSI), an estimate of bone strength, was obtained at a threshold of 280 mg/cm^3^ using cortmode 1. Bone strength index of compression (BSIc, g^2^/cm^4^) was derived at distal sites as the product of the total vBMD (g/cm^3^) squared and total CSA (cm^2^),^21^ obtained at the above thresholds.

Scans were qualitatively graded by radiographers through visual inspection to assess their suitability for analysis: scan slices with excessive movement or other artifacts and scout views with incorrect reference line placement were excluded. Calibration of the XCT 2000L system was performed using the manufacturer’s phantom: daily QA scans and weekly QC scans were performed throughout the study period to test scanner performance. Inter-operator precision was obtained from repeat scans from a subset of women (*n* = 28) with a root mean squared co-efficient of variation (RMS-CV%) between 0.8%-7.3% at the radius and 0.8%-4.8% at the tibia (see Table S1).

### Dual-energy X-ray absorptiometry

DXA scans were performed using the Hologic Discovery A instrument (Hologic Inc., Apex Version 13.4.2:3 software, S/N 83145) to measure whole body (WB) fat mass (kg and %). For practical reasons, the head was subtracted from the WB body scan regions. A single trained radiographer performed WB scans. RMS-CV for DXA body composition measurements, repeated twice in 30 participants, was 1.0% and 1.2% for lean mass and fat mass, respectively. Scanner performance over the period of the study was monitored using standard manufacturer QA and QC protocols.

### Ethical considerations

The study obtained ethical approvals from the Biomedical Research and Training Institute Institutional Review Board (Ref: AP152/2019) and the Harare Central Hospital Ethics Committee (Ref: HCHEC 181119/66), as well as the Medical Research Council of Zimbabwe (Ref: MRCZ/A/2551). Informed written consent was collected from all participants.

### Statistical analysis

**Figure 1:**
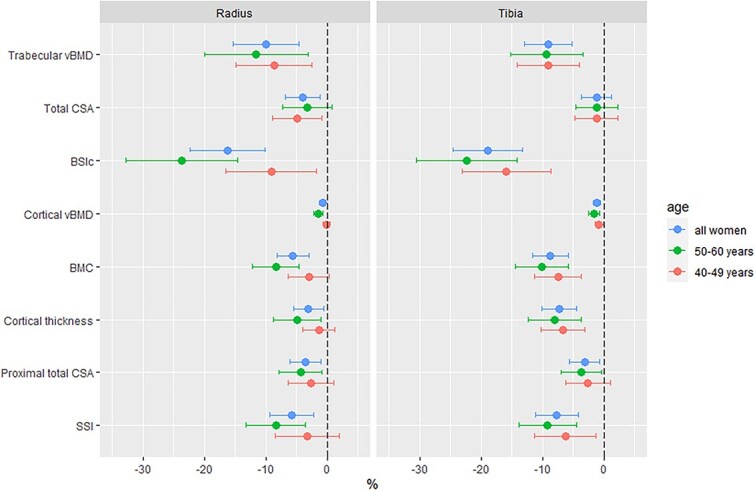
Symmetric percentage differences by HIV status in pQCT bone measures at the radius and tibia. Abbreviations: vBMD, volumetric BMD; CSA, cross-sectional area; BSIc, bone strength index of compression; SSI, stress–strain index.

All statistical analyses were performed using RStudio (2023.06.1 Build 524), R v4.3.1 (R Foundation for Statistical Computing, Vienna, Austria; https://www.r-project.org/). pQCT measures were distal radius and tibia trabecular vBMD, total CSA, and BSIc; proximal radius and tibia cortical vBMD, BMC, total CSA, cortical thickness, and SSI. Continuous descriptive data, where normally distributed, are tabulated as mean (SD), otherwise presented as median [IQR] for skewed data or as counts and percentages (*n*[%]) for categorical data (Table 1). pQCT data are tabulated as mean (SD) by HIV status and further stratified by age (40-49 and 50-60 yr) to investigate age interactions (Table S2). Differences by HIV status were investigated with independent sample *t*-tests, and Pearson’s Chi-squared tests for continuous and categorical variables, respectively (Table 1, Table S2). pQCT parameters were natural log (ln) transformed to allow for the visualization of between-group differences as symmetric percentages, that is, ln(a)-ln(b) multiplied by 100 is equivalent to the symmetric percentage difference between *a* and *b*, where *a* and *b* represent WLH and women without HIV, respectively (Figure 1).^22^^,^^23^

**Table 1:** Descriptive data for Zimbabwean women aged 40-60 by HIV status.

**HIV status**	**−ve**	**+ve**	***p*-value**
**Participant characteristics**	*n* = 193	*n* = 191	
**Age (yr)**	49.7 (5.8)	49.6 (5.8)	.918
**Age categories (*n* (%))** **40-49 yr** **50-60 yr**	94 (48.7) 99 (51.3)	96 (50.3) 95 (49.7)	.760^a^
**Menopause status (*n* (%))** **Pre-** **Peri-** **Post-**	89 (23.2) 26 (6.8) 78 (20.3)	76 (19.8) 24 (6.3) 91 (23.7)	.351^a^
**Highest level of education (*n* (%))** **None or primary only** **Secondary only** **Training college or university**	35 (18.1) 114 (59.1) 44 (22.8)	27 (14.1) 147 (77.0) 17 (8.9)	<.001^a^
**In employment (*n* (%))**	126 (65.3)	71 (37.2)	<.001
**Ever smoked/used tobacco (*n* (%))**	1 (0.5)	5 (2.6)	.099
**Drink alcohol (*n* (%))**	13 (6.7)	15 (7.9)	.675
**Anthropometry**			
**Height (m)**	1.62 (0.05)	1.61 (0.06)	.064
**Weight (kg)**	80.3 (16.2)	70.3 (14.8)	<.001
**Waist circumference**	91.4 (12.1)	86.9 (11.3)	<.001
**Hip circumference**	111 (12)	104 (12)	<.001
**BMI (kg/m** ^**2**^ **)**	30.7 (6.0)	27.2 (5.6)	<.001
**BMI categories (*n* (%))** **Underweight (<18.5 kg/m**^**2**^**)** **Normal (18.5 < 25 kg/m**^**2**^**)** **Overweight (25 < 30 kg/m**^**2**^**)** **Obese (≥30 kg/m**^**2**^**)**	0 (0) 36 (18.7) 61 (31.6) 96 (49.7)	6 (3.1) 58 (30.4) 74 (38.7) 53 (27.7)	<.001^a^
**Whole body fat mass (kg)**	32.4 (10.7)^(*n* = 192)^	26.7 (10.1)^(*n* = 190)^	<.001
**Whole body fat mass (%)**	41.9 (6.2)^(*n* = 192)^	39.3 (7.2)^(*n* = 190)^	–
**Fracture history**			
**Ever fractured (*n* (%))**	13 (6.7)	27 (14.1)	.018
**Major osteoporotic fracture (*n* (%))**	4 (2.1)	15 (7.9)	.009
**Reproductive characteristics**			
**Parity (*n* (%))** **Nulliparous (0)** **Low parity (1-3)** **Multiparous (≥4)**	2 (1) 113 (59) 78 (40)	8 (4) 121 (63) 62 (32)	.058
**Current contraceptive use (*n* (%))**	63 (32.6)	45 (23.6)	.048
**Hysterectomy (*n* (%))**	10 (5.2)	11 (5.8)	.804
**HIV characteristics**	*n*	Mean (SD)	Range
**Years since HIV diagnosis (yr)**	191	9.89 (5.93)	0-33.5
**Age at HIV diagnosis (yr)**	191	40.3 (7.1)	19.2-58.7
**Taking ART (*n* (%))**	191	182 (95.3)	–
**Duration on ART (yr)**	182	9.00 (4.53)	0.1-20.0
**Ever-used TDF**	182	153 (80.1)	–
**Viral load in copies/mL (*n* (%))** **<50** **≥50**	191	155 (81.2) 36 (18.8)	–

*p*-value from independent *t*-test (unpaired) *p* < .05 unless stated otherwise.

^a^Chi-squared test.

Menopause stage defined on time since last menstrual period: pre = regular menses; peri = irregular menses/amenorrhea ≤12 mo; post >12 mo after last menses. Independent *t*-tests (unpaired) used to explore between-group differences.

**Figure 2:**
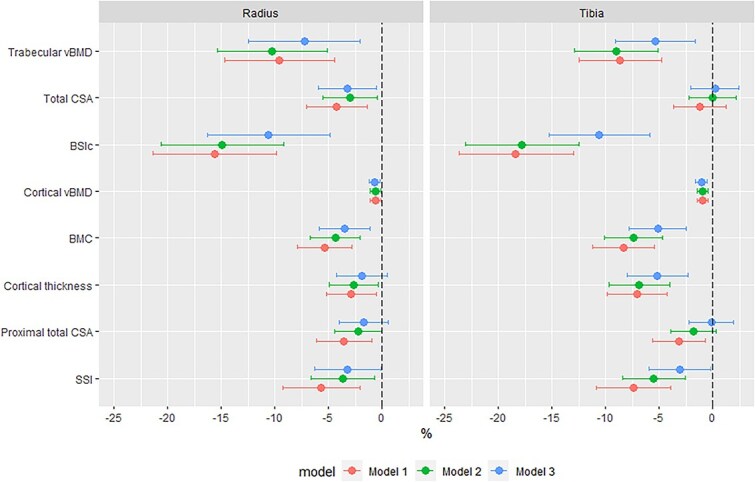
Estimates of differences in pQCT parameters at the radius and tibia by HIV status. Model 1 adjusted for age (yr) and menopause status (category); model 2 adjusted for age (yr) and menopause status (category), and height (m); and model 3 adjusted for age (yr) and menopause status (category), height (m), and fat mass (g). Abbreviations: vBMD, volumetric BMD; CSA, cross-sectional area; BSIc, bone strength index of compression; SSI, stress–strain index.

Linear regression was used to determine the relationships between HIV status and pQCT bone outcomes with a priori covariates to control for age and menopause status, in addition to anthropometry (height and weight) and body composition (fat mass), which may influence between-group comparisons. Three models were fitted: Model 1, adjusted for age (yr) and menopause status (pre-, peri-, and post-menopause); Model 2, with an additional adjustment for height (m); and Model 3, with an additional fat mass (kg) adjustment. Sensitivity analyses compared whether, (1) the substitution of weight for fat mass produced markedly different results in Model 3 or (2) the substitution of limb length for height in Model 3. Parity (*n*) and/or contraceptives were explored as potential explanatory variables. All pQCT parameters (dependent variable) were natural log transformed, to normalize their distribution and allow the expression of differences by HIV status (independent variable) as percentages (ie, the independent variable beta-coefficient*100 approximates the percentage difference per one unit change in the independent variable) (Figure 2, Table S3).^22^

Univariate logistic regression assessed associations between pQCT measures and MOF history. Beta coefficients were standardized to allow for the expression of the association per 1 SD change of each pQCT measure; log odds were exponentiated to produce odds ratios (OR) (Table 2).

**Table 2:** Unadjusted associations between pQCT measured bone parameters and self-reported history of major osteoporotic fracture in Zimbabwean women aged 40-60 yr. Data expressed as odds ratios and 95% CIs per SD increase in pQCT bone parameter and history of not sustaining a MOF.

***n* = 384**	**All women**		
**Radius 4%**	**OR**	**95% CI**	***p*-value**
**Trabecular vBMD**	1.09	0.68; 1.74	.723
**Total CSA**	1.41	0.86; 2.29	.172
**BSIc**	1.32	0.80; 2.17	.273
**Radius 33%**	OR	95% CI	*p*-value
**Cortical vBMD**	1.13	0.73; 1.76	.585
**Cortical BMC**	1.62	1.03; 2.57	.038
**Cortical thickness**	1.27	0.81; 1.97	.295
**Total CSA**	1.76	1.06; 2.91	.029
**SSI**	1.74	1.03; 2.93	.038
**Tibia 4%**	OR	95% CI	*p*-value
**Trabecular vBMD**	1.56	0.98; 2.50	.062
**Total CSA**	1.34	0.82; 2.18	.245
**BSIc**	2.19	1.26; 3.80	.005
**Tibia 38%**	OR	95% CI	*p*-value
**Cortical vBMD**	1.33	0.87; 2.01	.186
**Cortical BMC**	1.55	0.96; 2.51	.071
**Cortical thickness**	1.26	0.79; 1.99	.335
**Total CSA**	1.61	0.95; 2.75	.079
**SSI**	1.90	1.08; 3.35	.026

Nineteen women reported a previous history of major osteoporotic fracture (15 of whom were WLH).

Abbreviations: vBMD, volumetric BMD; CSA, cross-sectional area; BSIc, bone strength index of compression; SSI, stress–strain index.

**Figure 3:**
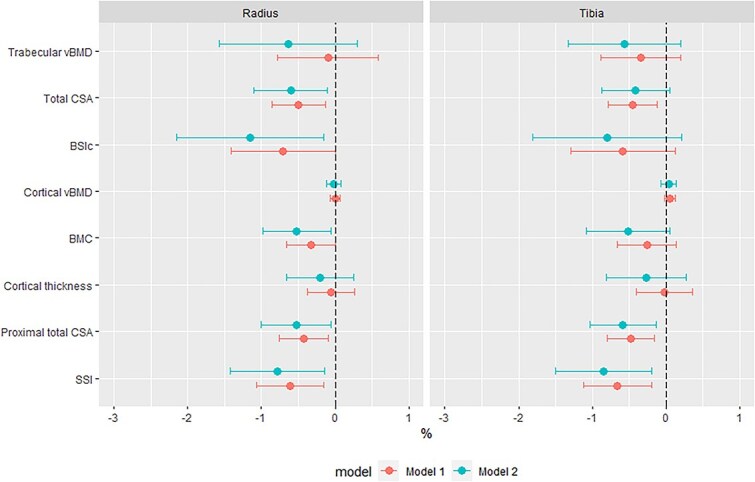
Associations between HIV duration and pQCT measured bone parameters in women living with HIV, as percentage difference in pQCT parameters per year living with HIV. Model 1, adjusted for years since HIV diagnosis, age and menopause status. Model 2, adjusted for years since HIV diagnosis, ART duration, age and menopause status. Abbreviations: vBMD, volumetric BMD; CSA, cross-sectional area; BSIc, bone strength index of compression; SSI, stress–strain index.

In WLH, linear regression was performed to determine the association between HIV duration (ie, years since diagnosis) and cortical and trabecular measures, adjusted for age and menopause status (Figure 3). Further adjustment for duration on ART was performed to explore if this attenuated the relationships between HIV duration and bone measures. Further, analysis of the potential impact of tenofovir disoproxil fumarate use (TDF, >80% exposed to TDF) or disease severity (<20% had a viral load ≥50 copies/mL) was precluded by the homogeneity of the WLH group.

## Results

### Descriptive statistics

Out of 399 women recruited, 393 had pQCT data for at least one radius or tibia scan site. Complete pQCT data were available for 384 (49.7% WLH) women (Figure S1). The 15 women (WLH, *n* = 5) who did not have complete pQCT data were younger (mean (SD) 47.2 (5.6) yr), taller (mean (SD) 1.64 (0.06) m), and more premenopausal than women with pQCT data. The proportion of women at each menopause stage was similar between WLH and HIV-negative women (Table 1). WLH had lower weight, BMI, fat mass, hip, and waist circumference compared with HIV-negative women (Table 1). WLH had a higher prevalence of self-reported fracture (both any fracture and MOF, Figure S2). Contraceptive use was less frequent in WLH compared with HIV negative women (23.6% vs. 32.6%, respectively, Table 1). Use of oral hormonal contraceptives was similar, and intrauterine device (IUD) and injectable (eg, medroxyprogesterone acetate) use was more common in HIV-negative women. The proportion of women who had had a hysterectomy was similar by HIV status, and all but one woman (in the HIV-negative group) had had a concurrent ovariectomy. No women reported taking menopausal hormonal therapy. Sociodemographic differences were apparent: WLH were less likely to be employed and had lower educational attainment. Alcohol and tobacco product use was low overall and not different between the groups. On average WLH had lived with a diagnosis of HIV for almost 10 yr and had taken ART for the majority of that time (Table 1). Most women (>95%) were on ART, of whom over 80% reported use of TDF (ever) and 42% reported dolutegravir use. 81% of WLH had a suppressed viral load defined as ≤50 copies/mL.

### Unadjusted differences in pQCT bone measures by HIV status and age

Absolute pQCT measures by HIV status and age stratification are presented as mean (SD) in Table S2 and summarized as mean percentage differences by HIV status in Figure 1. In all women, differences by HIV status were apparent for all pQCT bone outcomes except total CSA at the distal tibia: specifically, WLH had lower vBMD (total, trabecular, and cortical), thinner cortices, and lower estimated bone strength (both BSIc and SSI) compared to HIV-negative women (Figure 1 and Table S2). Age stratification demonstrated that differences in trabecular vBMD were of similar magnitude in younger (40-49 yr) and older (50-60 yr) women, though BSIc differences by HIV status were more pronounced in older WLH (Figure 1, Table S2). Cortical compartment deficits (vBMD, BMC, and thickness) were also more apparent in older WLH (Table S2, Figure 1). The magnitude of these cortical impairments was greatest at the load-bearing proximal tibia.

### Differences in pQCT bone measures adjusted for age, anthropometry, and fat mass by HIV status

Differences by HIV status, after adjustment for age and menopause status (Model 1), were similar to unadjusted results (Table S3). Further adjustment for height (Model 2) minimally reduced effect sizes for most outcomes and for all measures of bone geometry except distal radius CSA, attenuating differences by HIV status (Table S3). Controlling for fat mass in addition to height (Model 3) further reduced the magnitude of differences by HIV status (Table S3). Substituting weight for fat mass in Model 3 produced similar results (data not shown). Further adjustment for reproductive characteristics (parity and/or contraceptive use) did not influence the relationship between HIV and pQCT measures (data not presented). Adjusting for limb length rather than height produced broadly consistent between-group differences, though radius SSI was partially attenuated. Models 1-3 are summarized in Figure 2.

### Associations of pQCT measures and history of prior fracture

An increase of one SD in proximal radius BMC, total CSA, and SSI was associated with a lower risk of having sustained an MOF; distal tibia BSIc and proximal tibia SSI were also protective of prior history of MOF (Table 2). As only 4 HIV-negative women had sustained an MOF, stratification by HIV status was not performed.

### HIV-specific risk factors associated with deficits in cortical and trabecular bone

While time since HIV diagnosis was not associated with trabecular or cortical vBMD deficits at any site (Figure 3), at the distal radius and tibia each year since diagnosis was associated with a smaller CSA of −0.5 [−0.9; −0.1]% and −0.5 [−0.8; −0.1]%, respectively. Further adjustment for ART duration attenuated the association with tibia CSA, while radius CSA was robust to adjustment (Figure 3). Following adjustment for ART duration, distal radius BSIc was −1.2 [−2.2; −0.2]% lower for each year since diagnosis; however, no such association was seen at the distal tibia BSIc. At cortical-rich proximal sites, each year living with HIV was associated with lower total CSA (radius, −0.4 [−0.8; -0.1]%; tibia, −0.5 [−0.8; −0.2]%) and SSI (radius, −0.6 [−1.1; −0.2]%; tibia, −0.7 [−1.1; −0.2]%). Further adjustment for ART duration increased the effect size for these areal and strength measures, and each year since diagnosis became associated with a −0.5 [−1.0; −0.1]% lower radius BMC (Figure 3).

## Discussion

Midlife Zimbabwean WLH have substantial deficits in trabecular and cortical bone relative to their HIV-negative peers, as evidenced by lower trabecular and cortical vBMD of ~10% and ~1%, respectively. The magnitude of WLH’s lower trabecular vBMD was similar regardless of age, whereas cortical deficits in vBMD, BMC, and cortical thickness were more pronounced in older WLH. HIV-related deficits in vBMD, mass, and estimated strength were independent of age, menopause status, fat mass, and height, with estimates of strength approximately 10% and 3% lower in WLH at trabecular- and cortical-rich sites, respectively. These deficits were of similar magnitude at load-bearing and non-load-bearing sites. Further, in WLH we found for each year living with HIV, the strength estimates, BSIc and SSI, decreased by 1% at the radius, a common site of postmenopausal osteoporotic fracture. Taken together, these data suggest persistent deficits in vBMD, mass, and strength associated with HIV infection in midlife women.

These findings build on our previous DXA-based analysis, where WLH had lower aBMD and a higher prevalence of osteoporosis at all DXA sites (lumbar spine, femoral neck, or total hip) compared with HIV-negative women.^15^ In that analysis, osteoporosis was more prevalent at sites where a greater proportion of bone is trabecular (ie, lumbar spine > femoral neck > total hip), which is supported by our pQCT data. Broadly, our findings also align with work from neighboring South Africa, where a longitudinal study reported that HIV was associated with greater aBMD declines across the menopause transition.^14^ While the cross-sectional nature of our data precludes any assessment of change over time, stratification by age suggested different patterns of compartment-specific bone deficits with aging. This also suggests that trabecular deficits in WLH may predate the onset of menopause and may, to a large extent, drive the premenopausal loss of bone seen by DXA studies in South Africa and Uganda.^24–26^ Similarly, BSIc at distal sites calculated using total vBMD includes the cortical shell, which may explain why it follows the patterns seen at cortical-rich sites where deficits appear to occur later. While we sought to explore whether certain pQCT parameters were protective against fracture, this was only performed at the cohort level as fractures were uncommon with only 2% of HIV negative women reporting a major osteoporotic fracture compared to 8% of the WLH. Equally, in-depth analysis of the associations between HIV-specific factors and pQCT measures was hampered by the relative homogeneity of WLH—the majority on TDF and the majority virally suppressed, though notably below the 95% target.^27^ Perhaps unsurprisingly, each additional year living with HIV (since diagnosis) was found to be related to lower pQCT values at all sites, with the greatest influence seen in measures of bone area and strength. While adjustment for ART duration increased these effect sizes, this was modest, suggesting in this context ART use (predominantly TDF-containing regimens) did not contribute meaningfully to deficits in vBMD measurements in aBMD as seen in other cohorts, or that such deficits take a long time to accumulate. Unfortunately, given our cross-sectional study design, the relative similarities of years since diagnosis and duration of ART use, and a lack of information relating to regimen switching, we are unable to fully disentangle the effects of HIV, ART, or TDF on bone.

Few studies have explored potential HIV-related bone compartment-specific deficits in WLH, as to date most studies in WLH have used DXA. Only two HR-pQCT studies, from Canada (*n* = 100) and Switzerland (*n* = 44),^28^^,^^29^ in addition to our pQCT work (*n* = 430) from the Study of Women Entering and in Endocrine Transition (SWEET),^17^ have reported trabecular and cortical measures. The SWEET cohort comprised Black South African women living in urban Soweto aged 40-60 yr. However, as HIV was not a primary exposure in that study, the relatively small (*n* = 77) number of women known to be living with HIV and their distribution across menopause stages precluded formal analysis by HIV status. Despite this, the WLH had lower absolute pQCT bone measure values at each menopause stage compared to their peers, and within-group patterns of poorer bone health by menopause status appeared to be similar regardless of HIV status.^17^ Densitometric data across Southern Africa are scarce, which makes it interesting that South African women had much greater absolute vBMD and bone strength indices at both weight-bearing and non-weight-bearing sites than in the present study. There is a caveat in that the pQCT scanners are not cross-calibrated between the South African and current study, though similar scanning protocols were employed in both settings. A recent publication on global disparities in the prevalence of osteoporosis and osteopenia that included both the women in the HIV-negative arm of the present study and data from two cohorts in neighboring South Africa.^30^ Zimbabwean women had a 2- to 4-fold lower prevalence of osteoporosis than urban South African women, while rural South African women appeared to have an intermediate prevalence.^30^ Differences between Zimbabwean and South African women may in part be due to the greater body mass and weight of the South African women, particularly in urban settings, as well as differences in environmental and lifestyle factors.

Unsurprisingly, the Canadian and Swiss cohorts are more difficult to compare with our cohort, given differences in the nature of HIV epidemiology in North America and Europe, availability and use of different ART regimens, ethnicity, and age range. The mostly White (62%) Canadian cohort (WLH 31-69 yr; controls: 31-68 yr), who had lived with their HIV diagnosis for, on average, 20 yr, had bone deficits most pronounced in the trabecular compartment with a lasting negative impact of TDF use on trabecular bone.^28^ TDF was used by 49% (39% reported previous use and 12% never) in that Canadian study compared to >80% in the current Zimbabwean study, although for a shorter duration.^28^ Another potential explanation for this difference in compartment-specific findings may be imaging dependent, as the volume of interest (VOI) used (first-generation HR-pQCT) comprised a predominantly trabecular site at the epiphysis, whereas our cortical measures are from a diaphyseal cortical-rich site, albeit measured at lower resolution. In the Swiss study which compared premenopausal WLH to controls (aged median [IQR] 44.3 [38.9; 46.4] and 44.4 [41.3; 46.1] yr, respectively) the WLH had lived with a diagnosis of HIV for a median of 16.5 yr and only 45% were using TDF. WLH was reported to have lower tibial trabecular vBMD and trabecular number, and lower radius cortical vBMD in comparison to controls.^29^ No associations with TDF were reported, though again potentially due to the modest sample size.

### Strengths and limitations

These are unique data captured from an under-represented population, with a comparator group of similar age and menopause status. Our findings have some generalizability to the wider Southern African region, where the prevalence of chronic ART-controlled HIV in women at midlife remains high. However, the study has limitations; the study design is cross-sectional, so temporal directions of association cannot be established. Because of pandemic-associated lockdown restrictions, participants without HIV had to be identified and recruited through friendships of WLH, potentially introducing selection bias. Fracture history was captured by self-report and is subject to recall bias. Additionally, as fractures are rare events, our within-group analysis of the associations between pQCT parameters and fractures was underpowered. Details regarding ART usage were not sufficient to allow us to account for the potential impact of sequential regimes and switching, though it should be noted this is uncommon in this setting, and our findings are more likely related to unknown factors which impact poor HIV control.

## Conclusions

In Zimbabwean WLH, clear deficits in both trabecular and cortical compartments of the appendicular skeletons are present at mid-life compared with their HIV-negative peers. However, the magnitude of deficits was much greater in trabecular bone at non-load-bearing sites, which raises concerns for wrist fracture risk, as the distal radius is a common site of postmenopausal osteoporotic fracture. Our data also highlight that many of these deficits, particularly in trabecular bone, may originate closer to the time of diagnosis and therefore strategies to improve bone health in WLH may be required throughout the lifecourse and should not just target the menopause transition. This study adds to the growing literature from Southern Africa highlighting the urgent need to prioritize bone health in WLH both before and as they reach menopause. Only with further longitudinal studies in different cohorts of WLH across southern Africa will we be able to fully appreciate the lifecourse implications for fracture risk and determine context-specific preventive strategies to maintain bone health.

## Supplementary Material

Supplementary_materials_living_with_HIV_2024_08_20_Zjaf021

## Data Availability

The data used to prepare this manuscript are available from the senior author upon reasonable request.
