# Supplementary material for: Trabecular bone deficits predominate in the appendicular skeleton of midlife women living with HIV: findings from a cross-sectional study in Zimbabwe
Source: J Bone Miner Res. 2025 Jan 25;40(4):454–62. doi: 10.1093/jbmr/zjaf021 (PMC12010156; doi:10.1093/jbmr/zjaf021)
Supplement: Supplementary_materials_living_with_HIV_2024_08_20_Zjaf021 [file supplementary_materials_living_with_hiv_2024_08_20_zjaf021.docx]

**Supplementary materials**

**Figure S1.** Participant flow chart

**Table S1.** Root mean squared coefficients of variation for pQCT bone parameters and DXA body composition presented throughout the manuscript based on repeated scans from 30 women.

|  | | | | |
| --- | --- | --- | --- | --- |
|  | n | RMS CV% | n | RMS CV % |
|  | Radius 4% | | Tibia 4% | |
| Trabecular vBMD | 28 | 3.4 | 28 | 1.8 |
| Total CSA | 28 | 4.1 | 28 | 3.6 |
| BSIc | 28 | 6.9 | 28 | 4.8 |
|  | Radius 33% | | Tibia 38% | |
| Cortical vBMD | 28 | 0.8 | 28 | 0.8 |
| Cortical BMC | 28 | 3.5 | 28 | 1.5 |
| Cortical thickness | 28 | 3.1 | 28 | 1.5 |
| Total CSA | 28 | 4.6 | 28 | 3.0 |
| SSI | 28 | 7.3 | 28 | 4.1 |
|  | n | | RMS CV % | |
| Total body less head fat mass | 30 | | 1.2 | |
| vBMD = volumetric bone mineral density, CSA = cross-sectional area, BSIc = bone strength index of compression, BMC = bone mineral content, SSI = stress strain index. RMS = root mean squared | | | | |

**Table S2.** Unadjusted pQCT measured bone parameters for women living with HIV at mid-life and HIV-negative women aged 40-60 years in Zimbabwe

|  | | Overall | | | 40-49 years | | | 50-60 years | | |
| --- | --- | --- | --- | --- | --- | --- | --- | --- | --- | --- |
|  | | -ve | +ve | p-value | -ve | +ve | p-value | -ve | +ve | p-value |
| Site | pQCT outcome measures | n = 193 | n = 191 |  | 94 | 96 |  | 99 | 95 |  |
| Radius 4% | Trabecular vBMD (mg/cm^3^) | 167 (40) | 152 (41) | <0.001 | 172 (35) | 158 (37) | 0.012 | 162 (43) | 145 (43) | 0.008 |
|  | Total CSA (mm^2^) | 356 (47) | 342 (51) | 0.008 | 360 (47) | 343 (52) | 0.024 | 352 (48) | 341 (50) | 0.125 |
|  | BSIc (g^2^/cm^4^) | 0.37 (0.11) | 0.32 (0.10) | <0.001 | 0.38 (0.10) | 0.35 (0.09) | 0.014 | 0.36(0.11) | 0.29 (0.09) | <0.001 |
| Radius 33% | Cortical vBMD (mg/cm^3^) | 1194 (30) | 1185 (40) | 0.008 | 1204 (30) | 1202 (30) | 0.730 | 1184 (30) | 1167 (40) | <0.001 |
|  | Cortical BMC (mg/mm) | 93.5 (11.1) | 88.6 (12.0) | <0.001 | 95.6 (11.2) | 92.7 (10.6) | 0.072 | 91.5 (10.8) | 84.4 (12.0) | <0.001 |
|  | Cortical thickness (mm) | 2.55 (0.26) | 2.48 (0.32) | 0.022 | 2.61 (0.22) | 2.58 (0.25) | 0.347 | 2.50 (0.28) | 2.39 (0.36) | 0.020 |
|  | Total CSA (mm^2^) | 115 (14) | 112 (15) | 0.009 | 115 (14) | 112 (16) | 0.209 | 116 (14) | 111 (14) | 0.014 |
|  | SSI (mm^3^) | 253 (43) | 239 (44) | 0.002 | 253 (43) | 246 (47) | 0.269 | 252 (43) | 232 (40) | <0.001 |
| Tibia 4% | Trabecular vBMD (mg/cm^3^) | 204 (34) | 187 (38) | <0.001 | 208 (30) | 192 (37) | 0.001 | 199 (37) | 183 (39) | 0.003 |
|  | Total CSA (mm^2^) | 997 (112) | 988 (132) | 0.454 | 994 (102) | 987 (142) | 0.679 | 1000 (120) | 990 (122) | 0.524 |
|  | BSIc (g^2^/cm^4^) | 0.85 (0.22) | 0.71 (0.20) | <0.001 | 0.90 (0.20) | 0.77 (0.20) | <0.001 | 0.80 (0.23) | 0.65 (0.18) | <0.001 |
| Tibia 38% | Cortical vBMD (mg/cm^3^) | 1167 (30) | 1154 (40) | <0.001 | 1182 (20) | 1173 (30) | 0.033 | 1153 (30) | 1135 (40) | <0.001 |
|  | Cortical BMC (mg/mm) | 326 (42) | 300 (48) | <0.001 | 335 (42) | 311 (45) | <0.001 | 317 (40) | 288 (49) | <0.001 |
|  | Cortical thickness (mm) | 4.75 (0.60) | 4.43 (0.66) | <0.001 | 4.84 (0.58) | 4.53 (0.61) | <0.001 | 4.67 (0.61) | 4.33 (0.70) | <0.001 |
|  | Total CSA (mm^2^) | 424 (49) | 412 (55) | 0.020 | 425 (49) | 415 (61) | 0.240 | 424 (50) | 408 (48) | 0.028 |
|  | SSI (mm^3^) | 1750 (280) | 1630 (320) | <0.001 | 1783 (290) | 1685 (330) | 0.031 | 1718 (260) | 1574 (290) | <0.001 |
| pQCT parameters for WLH and HIV negative women expressed as Mean(SD), vBMD = volumetric bone mineral density, CSA = cross-sectional area, BSIc = bone strength index of compression, BMC = bone mineral content, SSI = stress strain index. Independent t-tests (unpaired) used to explore between-group differences. p-value from independent t-test (unpaired) | | | | | | | | | | |

**Table S3.** Estimated between-group differences, expressed as percentages, in pQCT bone measures between women living with HIV and HIV-negative women

| N=384 | Model 1 | | Model 2 | | Model 3 | |
| --- | --- | --- | --- | --- | --- | --- |
| Radius 4% | MD [95% CI] | p-value | MD [95% CI] | p-value | MD [95% CI] | p-value |
| Trabecular vBMD (mg/cm^3^) | -9.56% [-14.70%; -4.39%] | <0.001 | -10.3% [ -15.40%; -5.10%] | <0.001 | -7.26% [-12.50%; -2.04%] | 0.007 |
| Total CSA (mm^2^) | -4.22% [-7.06%; -1.37%] | 0.004 | -2.99% [-5.57%; -0.41%] | 0.023 | -3.25% [-5.93%; -0.57%] | 0.018 |
| BSIc (g^2^/cm^4^) | -15.6% [-21.40%; -9.87%] | <0.001 | -14.9% [-20.60%; -9.15%] | <0.001 | -10.6% [-16.30%; -4.85%] | <0.001 |
| Radius 33% | MD [95% CI] | p-value | MD [95% CI] | p-value | MD [95% CI] | p-value |
| Cortical vBMD (mg/cm^3^) | -0.63% [-1.13%; -0.14%] | 0.012 | -0.63% [-1.12%; -0.13%] | 0.013 | -0.71% [-1.22%; -0.19%] | 0.007 |
| Cortical BMC (mg/mm) | -5.37% [-7.89%; -2.84%] | <0.001 | -4.35% [-6.68%; -2.02%] | <0.001 | -3.49% [-5.88%; -1.09%] | 0.005 |
| Cortical thickness (mm) | -2.86% [-5.15%; -0.56%] | 0.015 | -2.67% [-4.97%; -0.37%] | 0.023 | -1.88% [-4.25%;0.50%] | 0.121 |
| Total CSA (mm^2^) | -3.56% [-6.13%; -0.98%] | 0.007 | -2.2% [-4.39%;0.00%] | 0.050 | -1.72% [-4.00%;0.56%] | 0.138 |
| SSI (mm^3^) | -5.66% [-9.24%; -2.08%] | 0.002 | -3.64% [-6.61%; -0.66%] | 0.017 | -3.21% [-6.30%; -0.12%] | 0.042 |
| Tibia 4% | MD [95% CI] | p-value | MD [95% CI] | p-value | MD [95% CI] | p-value |
| Trabecular vBMD (mg/cm^3^) | -8.66% [-12.50%; -4.82%] | <0.001 | -9% [-12.90%; -5.15%] | <0.001 | -5.39% [-9.11%; -1.67%] | 0.005 |
| Total CSA (mm^2^) | -1.23% [-3.71%;1.24%] | 0.327 | -0.04% [-2.21%;2.14%] | 0.974 | 0.17% [-2.09%;2.43%] | 0.880 |
| BSIc (g^2^/cm^4^) | -18.4% [-23.70%;-13.00%] | <0.001 | -17.8% [-23.10%;-12.50%] | <0.001 | -10.6% [-15.30%; -5.85%] | <0.001 |
| Tibia 38% | MD [95% CI] | p-value | MD [95% CI] | p-value | MD [95% CI] | p-value |
| Cortical vBMD (mg/cm^3^) | -0.96% [-1.49%; -0.42%] | <0.001 | -0.98% [-1.52%; -0.45%] | <0.001 | -1.08% [-1.64%; -0.53%] | <0.001 |
| Cortical BMC (mg/mm) | -8.34% [-11.20%; -5.47%] | <0.001 | -7.39% [-10.10%; -4.67%] | <0.001 | -5.16% [-7.84%; -2.48%] | <0.001 |
| Cortical thickness (mm) | -7.07% [-9.88%; -4.25%] | <0.001 | -6.87% [-9.70%; -4.05%] | <0.001 | -5.17% [-8.03%; -2.32%] | <0.001 |
| Total CSA (mm^2^) | -3.16% [-5.62%; -0.70%] | 0.012 | -1.82% [-3.90%;0.26%] | 0.086 | -0.15% [-2.21%;1.90%] | 0.883 |
| SSI (mm^3^) | -7.41% [-10.90%; -3.95%] | <0.001 | -5.52% [-8.43%; -2.60%] | <0.001 | -3.08% [-5.94%; -0.21%] | 0.035 |
| MD = mean difference; CI = confidence interval. MD (95% CI) with women without HIV as the reference group, such that negative values mean that those with HIV have lower values than those with HIV. vBMD = volumetric bone mineral density, CSA = cross-sectional area, BSIc = bone strength index of compression, BMC = bone mineral content, SSI = stress strain index.  Model 1 adjusted for age (years) and menopause status (category)  Model 2 adjusted for age (years) and menopause status (category), and height (m),  Model 3 adjusted for age (years) and menopause status (category), height (m), and fat mass (g).  Menopause stage defined on time since last menstrual period: pre = regular menses; peri = irregular menses/amenorrhea ≤12 months; post > 12 months after last menses. | | | | | | |
